# Alumina-Magnesia-Supported Ni for Hydrogen Production via the Dry Reforming of Methane: A Cost-Effective Catalyst System

**DOI:** 10.3390/nano13232984

**Published:** 2023-11-21

**Authors:** Abdulaziz A. M. Abahussain, Ahmed S. Al-Fatesh, Naitik Patel, Salwa B. Alreshaidan, Nouf A. Bamatraf, Ahmed A. Ibrahim, Ahmed Y. Elnour, Jehad K. Abu-Dahrieh, Ahmed E. Abasaeed, Anis H. Fakeeha, Rawesh Kumar

**Affiliations:** 1 Chemical Engineering Department, College of Engineering, King Saud University, P.O. Box 800, Riyadh 11421, Saudi Arabia; a.abahussain@ksu.edu.sa (A.A.M.A.); aidid@ksu.edu.sa (A.A.I.); aelnour@ksu.edu.sa (A.Y.E.); abasaeed@ksu.edu.sa (A.E.A.); anishf@ksu.edu.sa (A.H.F.); 2 Department of Chemistry, Indus University, Ahmedabad 382115, Gujarat, India; naitikvagdoda@gmail.com (N.P.); kr.rawesh@gmail.com (R.K.); 3 Department of Chemistry, Faculty of Science, King Saud University, P.O. Box 800, Riyadh 11451, Saudi Arabia; chem241@ksu.edu.sa (S.B.A.); 442204210@student.ksu.edu.sa (N.A.B.); 4 School of Chemistry and Chemical Engineering, Queen’s University Belfast, Belfast BT9 5AG, Northern Ireland, UK

**Keywords:** Al_2_O_3_, DRM, MgO, Ni catalyst, MgNiO_2_, cheap catalyst

## Abstract

5Ni/MgO and 5Ni/γAl_2_O_3_ are pronounced in the line of cheap catalyst systems for the dry reforming of methane. However, the lower reducibility of 5Ni/MgO and the significant coke deposition over 5Ni/γAl_2_O_3_ limit their applicability as potential DRM catalysts. The mixing capacity of MgO and Al_2_O_3_ may overcome these limitations without increasing the catalyst cost. Herein, a 5Ni/xMg(100 − x)Al (x = 0, 20, 30, 60, 70, and 100 wt. %) catalyst system is prepared, investigated, and characterized with X-ray diffraction, surface area and porosity measurements, H_2_-temperature programmed reduction, UV-Vis-IR spectroscopy, Raman spectroscopy, thermogravimetry, and transmission electron microscopy. Upon the addition of 20 wt. % MgO into the Al_2_O_3_ support, 5Ni/20Mg80Al is expanded and carries both stable Ni sites (derived through the reduction of NiAl_2_O_4_) and a variety of CO_2_-interacting species. CH_4_ decomposition at Ni sites and the potential oxidation of carbon deposits by CO_2_-interacting species over 5Ni/20Mg80Al results in a higher 61% H_2_-yield (against ~55% H_2_-yield over 5Ni/γAl_2_O_3_) with an excellent carbon-resistant property. In the major magnesia support system, the 5Ni/60Mg40Al catalyst carries stable Ni sites derived from MgNiO_2_ and “strongly interacted NiO-species”. The H_2_-yield over the 5Ni/60Mg40Al catalyst moves to 71%, even against a high coke deposition, indicating fine tuning between the carbon formation and diffusion rates. Ni dispersed over magnesia-alumina with weight ratios of 7/3 and 3/7 exhibit good resistance to coke. Weight ratios of 2/8 and 7/3 contain an adequate amount of reducible and CO_2_-interactive species responsible for producing over 60% of H_2_-yield. Weight ratio 6/4 has a proper coke diffusion mechanism in addition to achieving a maximum of 71% H_2_-yield.

## 1. Introduction

Global warming affects the entire ecological system through the rise in sea levels, drastic seasonal changes, and intensification of extreme weather events like heat waves, droughts, and wildfires. Greenhouse gases like CH_4_ and CO_2_ cause heat trapping in the atmosphere, resulting in global temperature rise. As a solution to this problem, the chemical, phot°Chemical, electr°Chemical, and catalytic conversion of CO_2_ into carbonates, oxazolidinones, ethanol, formic acid, methanol, dimethyl ether, and fuel [1,2,3,4,5,6] has drawn much attention to the use of CO_2_ as a resource. In the same way, the catalytic decomposition of CH_4_ also bears interest in fulfilling the clean energy H_2_ goal [7]. This time, the catalytic conversion of both greenhouse gases CH_4_ and CO_2_ together into hydrogen-rich syngas bears enormous hope towards diminishing global warming. This reaction is known as the dry reforming of methane (DRM). The product of DRM (H_2_-rich syngas) also has excellent synthetic and clean energy utility. From the catalytic point of view, DRM is a highly endothermic reaction (CH_4_ + CO_2_ → 2H_2_ + 2CO; Δ H^o^ = 247.34 kJ/mol), and it operates at high temperatures between 600 and 900 °C [8]. Pt, Pd, Ir, Ru, and Ni dispersed over MgO, CeO_2_, La_2_O_3_, Y_2_O_3_, TiO_2_, Si_2_O_3_, Al_2_O_3_, and ZrO_2_ are found to sustain such high temperatures and can catalyse the DRM reaction [9]. Cost-wise, metallic Ni dispersed over MgO, metallic Ni dispersed over Al_2_O_3_, and metallic Ni dispersed over a matrix of MgO-Al_2_O_3_ may be attractive. Alumina-supported Ni has been used frequently for DRM, but the rich surface acid profile of the catalyst triggers a high coke deposition, shading the active site and, ultimately inferior catalytic activity [10].

It is well accepted that metallic Ni is the active site for CH_4_ decomposition (into CH_x_ species; x = 0–3), and these CH_x_ species are further oxidized by CO_2_. If the oxidation of CH_x_ species is delayed, it may lead to severe carbon deposition over the active site, which may turn to inferior catalytic activity. The catalytic surface needs basicity for sound interaction with CO_2_ with support [11]. Group II metal oxides of the periodic table are known for their basicity. In the perovskite system (ABO_3_), A sites and B sites can be partially substituted by Mg to gain the inherent basic property. The partial substitution of Ni sites with Mg in LaNiO_3_ perovskite did not affect the thermal stability, but it enhanced the basicity [12]. LaNi_0.9_Mg_0.1_O_3_ catalyst had an enhanced basicity and metal–support interaction [12]. The partial substitution of La sites with Mg in LaNiO_3_ perovskite was also found to preserve the LaNiO_3_ and La_2_NiO_4_ phases in La_0.9_Mg_0.1_NiO_3_ catalysts [13]. Bai et al. found that, if Mg is added in a LaAlO_3_ support through the partial substitution of La, the “La_0.8_Mg_0.2_AlO_2.9_ supported Ni” catalyst induces the formation of more basic sites and oxygen vacancy [14]. Over acidic support like alumina, adding basic oxides like MgO, CaO, and BaO may be helpful for neutralizing the acidity of the support [15]. The promotional addition of these metal oxides over alumina-supported Ni enhances the reducibility in the order of Mg > Ba > Ca [16]. MgO bears basicity, high-temperature sustainability and the capability to stabilize metallic Ni by forming a MgO-NiO solid solution [17,18,19,20]. However, the calcination of Ni/MgO at temperatures beyond 700 °C and the rigid solid solution framework bring about a lower NiO reducibility, larger particle size, and inferior activity [18,19]. The solid-state reaction between MgO and zirconium hydroxide (ZrO_2_) has also been reported, which was found to stabilize the tetragonal phase of ZrO_2_ [21]. MgO also interacts strongly with silica and forms Mg-phyllosilicates [22], whereas it interacts with alumina and forms MgO-Al_2_O_3_ solid solutions [23], respectively. Ni stabilized over Mg-phyllosilicates/MgO-Al_2_O_3_ solid solution is highly dispersed.

Overall, the catalytic activity of 5Ni/MgO is limited by its lower reducibility, whereas the performance of the 5Ni/γAl_2_O_3_ catalyst is limited by its high acidity [10,18,19]. It is necessary to think between these two extremes, because one is the solution to another. The solid solution formation capability between “MgO and Al_2_O_3_” allows one to mix two metal oxides to a great extent. From the Ni/γAl_2_O_3_ side, the extent of MgO (basic) addition to acidic Al_2_O_3_ needs to be optimized to overcome the harmful effect of acidity towards DRM. Herein, to justify this hypothesis, Ni catalysts loaded on different magnesia modified alumina supports, 5Ni/xMgO(100 − x)Al_2_O_3_ (x = 100, 70, 60 wt. %), were prepared and tested for the DRM reaction. Again, from the 5Ni/MgO side, the extent of Al_2_O_3_ addition along with MgO needs to be optimized to enhance the reducibility for the optimum catalytic performance. To justify this hypothesis, 5Ni/xMgO(100 − x)Al_2_O_3_ (x = 30, 20, 0 wt. %) catalysts were prepared and investigated for DRM. These catalyst systems were characterized thoroughly via X-ray diffraction, surface area and porosity measurements, a H_2_-temperature programmed reduction, UV-Vis spectroscopy, infrared spectroscopy, Raman spectroscopy, thermogravimetry, and transmission electron microscopy. The novelty of this work is to study the modifications in crystallinity, surface parameters, reducibility, electronic transition, the population of CO_2_-interating species, and type of carbon deposit over magnesia-alumina supported Ni catalysts which are prepared with different weight ratios of magnesia and alumina as supports and 5 wt. % Ni as active sites. The fine correlation of the characterization results with the catalytic activity of 5Ni/xMgO(100 − x)Al_2_O_3_ (x = 100, 70, and 60 wt. %) and 5Ni/xMgO(100 − x)Al_2_O_3_ (x = 30, 20, 0 wt. %) catalysts is aimed to open the way for developing a robust DRM catalyst system by using cheap chemicals like MgO, Al_2_O_3_, and nickel nitrate.

## 2. Materials and Methods

### 2.1. Materials

The following materials were used in the catalyst preparation: Nickel nitrate hexahydrate [Ni(NO_3_)_2_·6H_2_O, 98%, Alfa Aesar, St. louis, Mo, USA], Alumina (Al_2_O_3_; SASOL Anckelmannsplatz 1, 20,537, Hamburg, Germany), and Magnesium oxide (MgO; SASOL Anckelmannsplatz 1, 20,537, Hamburg, Germany).

### 2.2. Catalyst Preparation

Ni supported over alumina or magnesia or magnesia-alumina was prepared by mixing a nickel nitrate precursor solution (equivalent to 5 wt. % Ni) with alumina, magnesia, and magnesia-alumina (equivalent to x wt. % magnesia and (100 − x) wt. % alumina; x = 0, 20, 30, 60, 70, and 100) supports followed by drying at 80 °C for 30 min and calcining at 600 °C for three hours. The catalysts are abbreviated as 5Ni/γAl_2_O_3_, 5Ni/MgO, and 5Ni/xMg(100 − x)Al (x = 20, 30, 60, and 70 wt. %).

### 2.3. Catalyst Characterization

The X-ray Diffraction study of the catalyst sample was carried out with a Miniflex Rigaku diffractometer (Rigaku, Saudi Arabia) using a Cu Kα radiation source operated at 40 kV and 40 mA. The phase analysis of the diffraction patterns was performed using the X’pert high score assisted with the JCPDS database. The catalyst sample’s adsorption isotherms, surface area, pore volume, and pore diameter were obtained from the Micromeritics Tristar II 3020 instrument (Micromatics, Fridley, MN, USA). The H_2_-Temperature Programmed Reduction (H_2_-TPR of catalyst sample (70 mg)) was taken by Micromeritics Aut°Chem II 2920 (Micromatics, USA) equipped with a thermal conductivity detector (TCD) by using a mixture of 10% H_2_/Ar at a flow rate of 40 mL/min, from a temperature range from 50 °C to 1000 °C (heating ramp 10 °C/min). CO_2_-temperature programmed desorption (CO_2_-TPD) was carried out over Micromeritics Aut°Chem II 2920 (Micromatics, USA). A 10% CO_2_/He gas mixture (flow rate 30 mL/min) was passed over the catalyst samples (70 mg) at 50 °C for 30 min. Further, the temperature of the catalyst bed was raised to 900 °C. During this temperature rise, CO_2_ was desorbed from the sample and the conductivity was recorded by TCD. The Fourier transform infrared (FTIR) spectra and ultraviolet-visible spectra of catalyst samples were taken by Prestige-21 SHIMADZU and V-570 (JASCO, Oklahoma City, OK, USA), respectively. A thermo-gravimetric (TGA) analysis of the spent catalyst samples (10–15 mg) was carried out by Shimadzu TGA-51 (Shimadzu Corporation, Kyoto, Japan) in the temperature range from room temperature to 1000 °C (at 20 °C/min temperature ramp) under O_2_. The mass change in the spent catalyst sample was constantly monitored during heat treatment. The Raman spectra of the spent catalysts were taken by a Laser Raman (NMR-4500) Spectrometer (JASCO, Tokyo, Japan) in a wavenumber range of 1200–1800 cm^−1^ under a 532 nm excitation beam, 1.6 mW laser intensity, and 10 s exposure time at 3 accumulations. The catalyst morphology was taken by a 120kV JEOL JEM-2100F (Akishima, Japan) transmission electron microscope.

### 2.4. Catalytic Activity Test

The dry reforming of the methane reaction was performed over 0.1 g of catalyst packed in a continuous-flow, fixed-bed reactor (9.0 mm internal diameter and 300 mm length) (PID Eng. And Tech., Alcovendas, Spain) at atmospheric pressure. Axially aligned thermocouple were used to measure the temperature of the catalyst bed. Before the DRM reaction, the catalyst was reduced under H_2_ (flow rate 30 mL/min) at 800 °C for one hour. Subsequently, to remove any residual H_2_, the reactor was purged with N_2_ (flow rate 20 mL/min) at 800 °C for 60 min. The reactant feed gas, CH_4_/CO_2_/N_2_, with a volume ratio of 30/30/10 (total flow rate 70 mL/min; space vel°City 42,000 mL/(h·g_cat_)) ©,s was allowed to pass through the catalyst at a 700 °C reaction temperature. The reaction products and unconverted feed gases from the reactor were evaluated quantitatively by using an online GC (GC-Shimadzu 2014) equipped with a molecular sieve 5A and Porapak Q column and thermal conductivity detector (TCD). The H_2_ yield (%) and CO yield (%) of the reaction were calculated as shown below.

$$H_{2}\;\mathrm{yield}\;(\%)=\frac{Mole\;of\;H_{2}\;in\;Product}{2\times Mol\;of\;{{CH}_{4}}_{in}}\times 100\%$$

$$\mathrm{CO}\;\mathrm{yield}\;(\%)=\frac{Mole\;of\;CO\;in\;Product}{Mol\;of\;{{CH}_{4}}_{in}+Mol\;of\;{{CO}_{2}}_{in}}\times 100\%$$

## 3. Results & Discussion

### 3.1. Characterization Results

**Figure 1:**
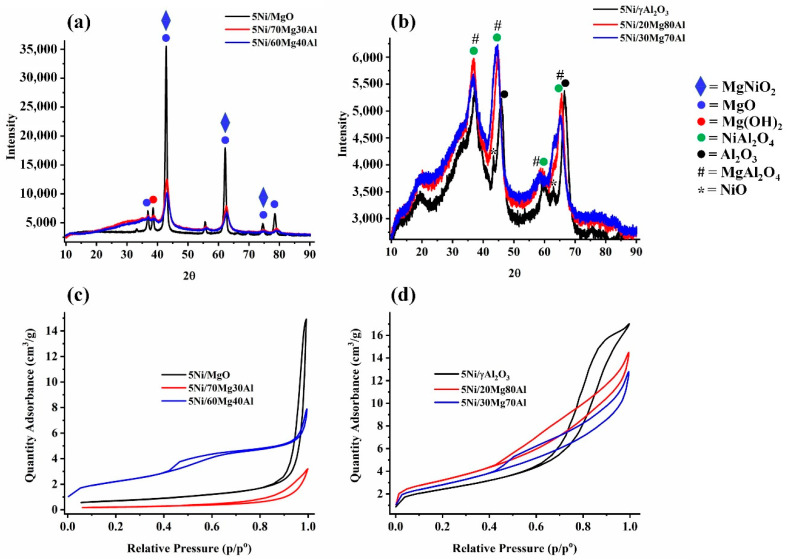
X-ray diffraction pattern of (**a**) 5Ni/MgO and 5Ni/xMg(100 − x)Al (x = 70, 60 wt. %) (**b**) 5Ni/γAl_2_O_3_ and 5Ni/xMg(100 − x)Al (x = 20, 30 wt. %); Nitrogen sorption isotherms of (**c**) 5Ni/MgO and 5Ni/xMg(100 − x)Al (x = 70, 60 wt. %) (**d**) 5Ni/γAl_2_O_3_ and 5Ni/xMg(100 − x)Al (x = 20, 30 wt. %).

The X-ray diffraction patterns of the 5Ni/MgO, 5Ni/70Mg30Al, and 5Ni/60Mg40Al catalysts are shown in Figure 1a. 5Ni/MgO had higher-intensity diffraction patterns than 5Ni/γAl_2_O_3_, indicating more crystallinity earlier than later. The 5Ni/MgO catalyst had a cubic MgNiO_2_ phase (at 2θ = 42.9°, 62.2°, and 74.6°; JCPDS reference number 00-003-0999), cubic MgO phase (at 2θ = 36.8°, 42.9°, 62.2°, 74.6°, and 78.5°; JCPDS reference number 01-071-1176), and hexagonal Mg(OH)_2_ phase (at 2θ = 38.6°, and 62.2°; JCPDS reference number 00-001-1169) [24]. Upon incorporating alumina in the major magnesia support (5Ni/70Mg30Al and 5Ni60Mg40Al catalysts), the intensity of these peaks was decreased markedly. The X-ray diffraction patterns of 5Ni/γAl_2_O_3_, 5Ni/20Mg80Al, and 5Ni/30Mg70Al are shown in Figure 1b. The alumina-supported Ni catalyst had an individual cubic NiO phase (at 2θ = 37.1°, 43.4°, and 63°; JCPDS reference number 00-047-1049), cubic Al_2_O_3_ phase (at 2θ = 39.4°, 45.8°, and 66.5°; JCPDS reference number 00-001-1303), and mixed cubic NiAl_2_O_4_ phase (at 2θ = 37.1°, and 59.6°; JCPDS reference number 00-001-1299). Upon incorporating magnesia proportions in the major support alumina (5Ni/20Mg80Al and 5Ni30Mg70Al), individual metal oxide peaks like a cubic Al_2_O_3_ phase and cubic NiO disappeared, as well as mixed oxide peaks like a cubic NiAl_2_O_4_ (at 2θ = 37.1°, 45.1°, and 65.5°; JCPDS reference number 00-001-1299) and cubic MgAl_2_O_4_ phase (at 2θ = 37.1°, 45.1°, 58.9°, and 65.5°; JCPDS reference number 01-084-0377) appeared.

The N_2_ adsorption–desorption isotherms of the catalysts are shown in Figure 1c,d. All isotherms are characterized by a hysteresis loop or Type IV isotherms. Over 5Ni/MgO, a sharp inflection at a relative pressure of 0.85 and a H1-type hysteresis loop are found, which indicate the uniform distribution of cylindrical pores (average diameter 35.5 nm) (Table 1). Upon incorporating 30 wt. % alumina in the major support magnesia (5Ni/70Mg30Al), the surface area, pore size, and pore volume dropped spontaneously. The hysteresis loop pattern was also changed to H3, indicating a change of the pore type from cylindrical to plat-like (non-rigid aggregate pore) mesopores [25]. Upon further increasing the proportion of alumina in the magnesia-alumina support, the 5Ni/60Mg40Al catalyst showed an excellent improvement in surface area and modification in the pore type. It had a H4 hysteresis loop, which confirms the presence of both micropores and mesopores. Over the 5Ni/γAl_2_O_3_ catalyst, a H1 hysteresis loop was found again, indicating ordered cylindrical mesopores. Upon incorporating 20 wt. % magnesia in the major alumina support (5Ni/20Mg80Al), the surface area of the catalyst increased by 33%, and the hysteresis loop pattern changed from H1 to H3. It indicates the expansion of the framework, as well as the presence of plate-like mesopores upon incorporating magnesia proportions with alumina as a support. However, upon further increasing the proportion of magnesia in the magnesia-alumina-supported Ni catalyst (5Ni/30Mg70Al), the surface area decreased markedly and the H3 hysteresis loop was retained. The pore size distribution plot (dV/dlogW vs. W) of 5Ni/MgO, 5Ni/xMg(100 − x)Al (x = 70, 60, 30, 20 wt. %) is shown in Figure S1. The 5Ni/γAl_2_O_3_ catalyst shows a monomodal pore size distribution with a pore diameter of approximately 12 nm [26]. The rest of the catalysts belong to a multimodal distribution of pore size, where pores in a macroporous region are also prominent.

**Figure 2:**
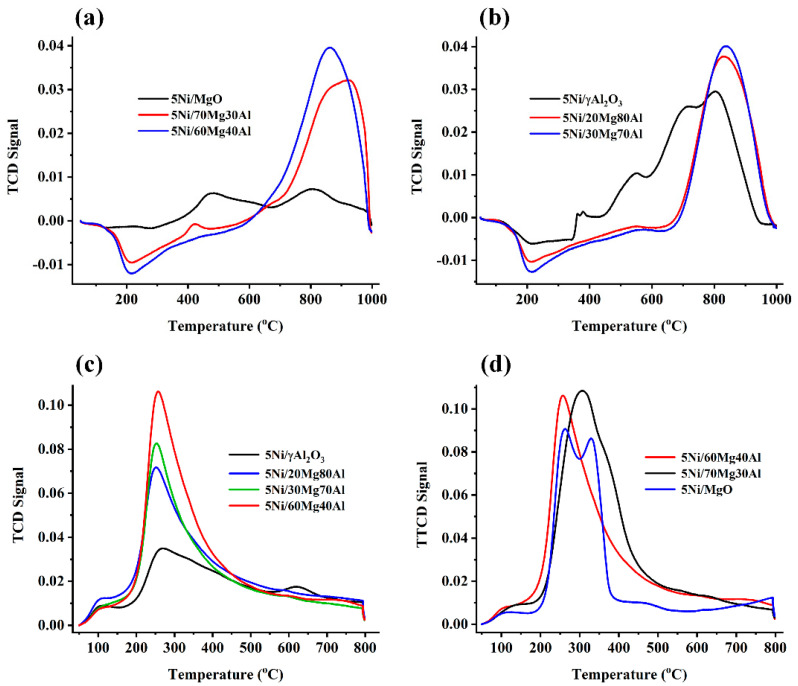
H_2_−Temperature programmed reduction of (**a**) 5Ni/MgO and 5Ni/xMg(100 − x)Al (x = 70, 60 wt. %) (**b**) 5Ni/γAl_2_O_3_ and 5Ni/xMg(100 − x)Al (x = 20, 30 wt. %)s; CO_2_−Temperature programmed deadsorption of (**c**) 5Ni/γAl_2_O_3_ and 5Ni/xMg(100 − x)Al (x = 20, 30,60 wt. %) (**d**) 5Ni/MgO and 5Ni/xMg(100 − x)Al (x = 70, 60 wt. %).

The H_2_-TPR of the catalyst samples are shown in Figure 2. The 5Ni/MgO catalyst showed reduction peaks at 474 °C and 800 °C (Figure 2a). The former is attributed to the reduction of NiO species, which moderately interacted with the support, and the later one (reduction peak at 800 °C) is attributed to the reduction of Ni^2+^ species from mixed oxide into the metallic Ni form [27]. The XRD results indicate the presence of a cubic MgNiO_2_ phase. Liu et al. found that, upon reduction, MgNiO_2_ generates Ni clusters with a size range of 7–9 nm [28]. Overall, it can be said that the H_2_-TPR peak of about 800 °C is attributed to the reduction of MgNiO_2_ species into metallic Ni. It is also noticeable that we carried out reductive pre-treatment of the catalyst under H_2_ before the DRM reaction at 800 °C. Reductive pretreatment aims to create a catalytic active site metallic Ni before the DRM reaction. Upon the reductive pretreatment at 800 °C, all the catalytic active sites (metallic Ni) for the DRM reaction were generated. Upon the incorporation of 30 wt. % alumina in a major magnesia support (5Ni/70Mg30Al), the reduction peak of about 800 °C was significantly magnified. However, the XRD diffraction patterns showed a fall in the MgNiO_2_ phase’s intensity in the 5Ni/70Mg30Al catalyst. This indicates that the reduction peak at 800 °C was not only due to the reduction of MgNiO_2_, but also due to the reduction of NiO species, which strongly interacted with the support [11]. Overall, it can be said that the incorporation of alumina in a magnesia support brings about a strong interaction of NiO species. After the reduction under H_2_, a high density of metallic Ni (derived from the reduction of “strongly interacted NiO species) remains present during the DRM reaction over the 5Ni/70Mg30Al catalyst. The growth of “strongly interacted NiO species” on the catalyst’s surface is continued over the 5Ni/60Mg40Al catalyst. The alumina-supported Ni catalyst shows reduction peaks for weak, moderately, and strongly interacted NiO species at ~400 °C, 450–700 °C, and ~800 °C, respectively (Figure 2b). Strongly interacted NiO-species are attributed to NiAl_2_O_4_, which generates stable metallic Ni particles after reductive treatment [29]. Interestingly, upon the incorporation of magnesia in the major alumina-supported Ni catalyst, only reducible “strongly interacted NiO-species” developed. So, the 5Ni/20Mg80Al and 5Ni/30Mg70Al catalysts had a single intense reduction peak at 800 °C. The CO_2_-desoprtion profile of the magnesia-alumina supported Ni catalyst existed in the 200–450 °C temperature range, which is attributed to moderate strength basic sites/surface oxygen [30] (Figure 2c,d). Ni/γAl_2_O_3_ had a minimum CO_2_ desorption acquiring a minimum basicity. The proportion of Mg was increased (upto 60 wt. % Mg) in the magnesia-alumina supported Ni catalyst system, and the basicity of the catalyst was increased. Upon 70 wt. % magnesia incorporation, the intensity of the CO_2_ desorption peak increased and broadened towards a higher temperature. It indicate the organization of a relatively stronger basic site over the 5Ni/70Mg30Al catalyst than the rest of the catalysts. However, over the magnesia-supported Ni catalyst, the peak intensities were relatively decreased and split into two peaks. 5Ni/MgO had moderate strength basic sites, but the highest amount of basic sites were organized over the magnesia-alumina (70:30 w ratio)-supported Ni catalyst.

**Figure 3:**
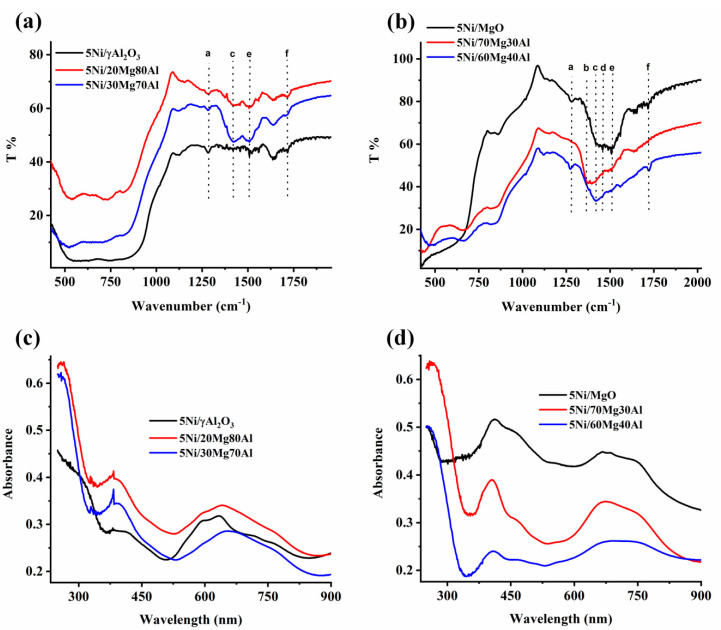
Infrared spectra of (**a**) 5Ni/γAl_2_O_3_ and 5Ni/xMg(100 − x)Al (x = 20, 30 wt. %) (**b**) 5Ni/MgO and 5Ni/xMg(100 − x)Al (x = 70, 60 wt. %); Ultraviolet−visible spectra of (**c**) 5Ni/γAl_2_O_3_ and 5Ni/xMg(100 − x)Al (x = 20, 30 wt. %) (**d**) 5Ni/MgO and 5Ni/xMg(100 − x)Al (x = 70, 60 wt. %).

The infrared spectra of the catalyst samples from 1000 to 2000 cm^−1^ are informative regarding CO_2_-interacting surface species (Figure 3a,b). The peaks at 1270 cm^−1^ and 1730 cm^−1^ are attributed to bidentate carbonate (shown by a, f dotted lines in Figure 3a,b) [10,31], whereas the peaks about 1384 cm^−1^ and 1517 cm^−1^ are attributed to unidentate carbonate species (shown by b,e dotted lines in Figure 3a,b) [32,33]. IR vibrational peaks at about 1417 cm^−1^ for carboxylate species and about 1460 cm^−1^ for CO_3_^2−^ species are also noticed (shown by c, d dotted lines in Figure 3a,b) [31,32,33]. The alumina-supported Ni catalyst has only diffuse peaks for bidentate carbonate species (indicated by a, f dotted lines in Figure 3a). Upon the incorporation of 20 wt. % MgO in the major alumina support (5Ni/20Mg80Al), peaks for carboxylate and unidentate carbonate also appear (shown by c,e dotted lines in Figure 3a). Upon increasing the MgO proportion in the magnesia-alumina supported Ni catalyst (5Ni/30Mg70Al), the intensity of these peaks grows. Interestingly, when the support is made up majorly by magnesia (5Ni/MgO, 5Ni/70Mg30Al, and 5Ni/60Mg40Al catalysts), the catalytic surfaces are enriched with more CO_2_-interacting species than the 5Ni/30Mg70Al and 5Ni/20Mg80Al catalysts (Figure 3b). The UV-Vis spectroscopy of the catalyst sample shows a charge transfer transition from O^2−^ to Ni^2+^ (in °Ctahedral coordination) at a lower wavelength (260 nm), the d-d transition from ^2^A_2g_ (F) to ^3^T_1g_ (P) state of Ni^2+^ (°Ctahedral coordination) at a 410 nm wavelength, and d-d transition from ^3^T_1_(F) to the ^3^T_1_ (P) state of Ni^2+^ in a tetrahedral coordination at a 634 nm wavelength (Figure 3c,d) [34]. It is found that the charge transfer band from O^2−^ to Ni^2+^ is intensified when the dual-metal-oxide-supported Ni catalysts are used as a support. The d-d transition band from ^2^A_2g_ (F) to the ^3^T_1g_ (P) state of Ni^2+^ (°Ctahedral coordination) is also intensified in the 5Ni/20Mg80Al and 5Ni/30Mg70Al catalysts. But both types of d-d transition band (discussed above) are relatively suppressed in the 5Ni/70Mg30Al and 5Ni/60Mg40Al catalysts.

**Figure 4:**
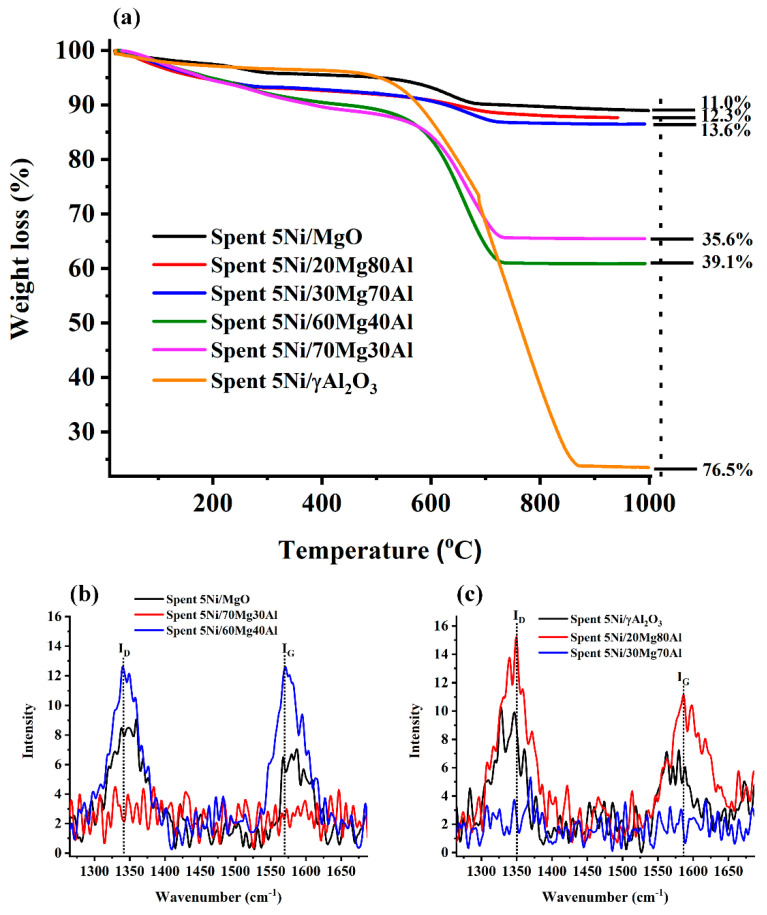
(**a**) Thermogravimetry analysis of spent 5Ni/MgO, spent 5Ni/xMg(100 − x)Al (x = 20, 30, 60, 70 wt. %) and spent 5Ni/γAl_2_O_3_ catalysts, (**b**) Raman spectra of spent 5Ni/MgO and spent 5Ni/xMg(100 − x)Al (x = 70, 60 wt. %) catalysts, and (**c**) Raman spectra of spent 5Ni/γAl_2_O_3_ and spent 5Ni/xMg(100 − x)Al (x = 20, 30 wt. %) catalysts.

The thermogravimetry results of the catalysts are shown in Figure 4a. Interestingly, the 5Ni/MgO catalyst has the least coke deposition (11% weight loss), whereas the 5Ni/γAl_2_O_3_ catalyst has the highest coke deposition (76.5% weight loss). The 5Ni70Mg30Al and 5Ni60Mg40Al catalysts enhance the coke deposition, giving a 35.6% and 39.1% weight loss, respectively. It seems that, upon increasing the proportion of acidic alumina, CH_4_ decomposition (into CH_x_) excels over CH_x_ oxidation, resulting in the oligomerization of CH_x_ or the formation of coke deposit over the catalyst surface. In the same line, it is found that, with the incorporation of basic magnesia along with acidic alumina as a support, the coke deposition over the 5Ni20Mg80Al and 5Ni30Mg70Al catalysts is greatly delayed. Upon increasing basic MgO into acidic Al_2_O_3_, the acidity of the catalyst is neutralized and the basicity rises, which turns into the interaction of a more significant number of CO_2_ for the oxidation of carbon deposits. So, 5the Ni20Mg80Al and 5Ni30Mg70Al catalysts have just a 12.3% and 13.6% weight loss. To understand the type of carbon deposit, the RAMAN spectra of spent catalyst samples were taken (Figure 4b,c). The Raman spectra of spent catalysts are characterized by a broad peak at about 1340 cm^−1^ for the defect carbon band (I_D_) and about 1570 cm^−1^ for the graphite carbon band (I_G_) [35]. Interestingly, the 5Ni/70Mg30Al and 5Ni/30Mg70Al catalysts have a deficit of both the defect carbon band and graphitic carbon band (Figure 4b,c). However, upon increasing the proportion of alumina in both catalyst types (5Ni/60Mg40Al and 5Ni/20Mg80Al), the peak intensity of these peaks (I_D_ and I_G_) reach the maximum in its group (Figure 4b,c). It indicates that Ni dispersed over MgO-Al_2_O_3_ with a weight ratio of 7/3 or 3/7 shows a strong suppression for defect and graphitic carbon deposits during the DRM reaction. The ratio of band intensities I_G_/I_D_ represents the graphitic degree of coke [36]. The I_D_/I_G_ ratios of 5Ni/MgO, 5Ni/60Mg40Al, 5Ni/20Mg80Al, and 5Ni/γAl_2_O_3_ are found to be 0.71, 1.01, 0.69, and 0.63, respectively. Over the 5Ni/60Mg40Al catalyst, almost equal amounts of defect carbon and graphitic carbon band are found.

**Figure 5:**
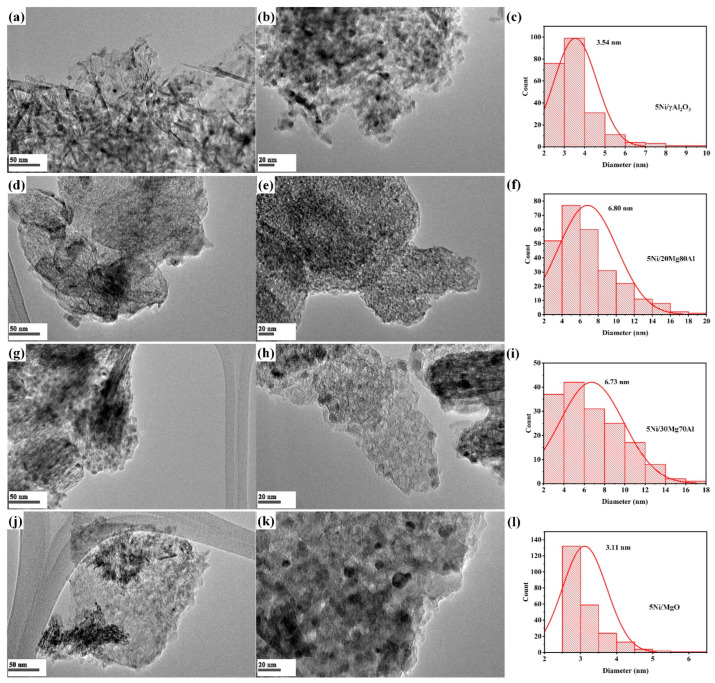
TEM image of (**a**) 5Ni/γAl_2_O_3_ on 50 nm scale, (**b**) 5Ni/γAl_2_O_3_ on 20 nm scale, (**d**) 5Ni/20Mg80Al on 50 nm scale, (**e**) 5Ni/20Mg80Al on 20 nm scale, (**g**) 5Ni/30Mg70Al on 50 nm scale (**h**) 5Ni/30Mg70Al on 20 scale, (**j**) 5Ni/MgO on 50 nm scale, and (**k**) 5Ni/MgO on 20 nm scale; particle size distribution of (**c**) 5Ni/γAl_2_O_3_, (**f**) 5Ni/20Mg80Al, (**i**) 5Ni/30Mg70Al, and (**l**) 5Ni/MgO.

The transmission electron microscopy of the catalyst and Ni particle size distribution of the 5Ni/MgO, Ni/xMg(100 − x)Al (x = 20, 30 wt. %) and 5Ni/γAl_2_O_3_ catalysts are depicted in Figure 5. The particle size over the 5Ni/γAl_2_O_3_ and 5Ni/MgO catalyst are found to be 3.54 nm and 3.11 nm (Figure 5c,l). Interestingly, the Ni particle dispersed over the dual-metal-oxide supports are found to be larger. The particle size of Ni over 5Ni/20Mg80Al and 5Ni/30Mg70Al are found to be 6.80 nm and 6.73 nm, respectively (Figure 5f,i).

### 3.2. Catalytic Activity Results and Discussion

**Figure 6:**
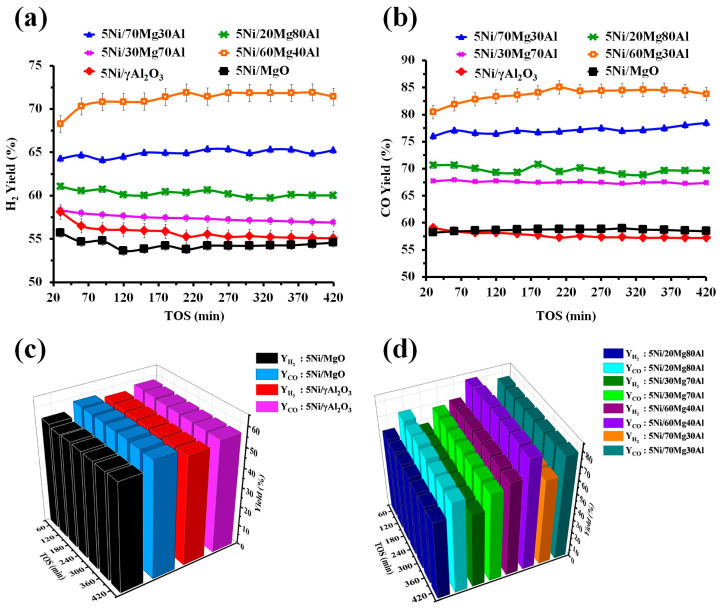
Catalytic activity results: (**a**) H_2_ yield (%) versus time on stream of 5Ni/MgO, 5Ni/xMg (100 − x)Al (x = 20, 30, 60, 70 wt. %) and 5Ni/γAl_2_O_3_ catalysts, (**b**) CO yield (%) versus time on stream of 5Ni/MgO, 5Ni/xMg (100 − x)Al (x = 20, 30, 60, 70 wt. %) and 5Ni/γAl_2_O_3_ catalysts, (**c**) “H_2_ yield (%) and CO yield (%)” versus time on stream of 5Ni/MgO and 5Ni/γAl_2_O_3_ catalysts, and (**d**) “H_2_ yield (%) and CO yield (%)” versus time on stream of 5Ni/xMg (100 − x)Al (x = 20, 30, 60, 70 wt. %) catalysts.

The H_2_-Yield versus time on stream and CO-yield versus time on stream profiles of the different catalyst systems are shown in Figure 6a,b. The comparative plot of “H_2_-yield and CO-yield” versus time on stream for different catalysts is also shown in Figure 6c,d. The activity experiments are reproduced thrice for all the catalysts. The error of catalytic activity is between 2 and 4% for all the experiments. The 5Ni/MgO and 5Ni/γAl_2_O_3_ catalysts have Ni containing mixed-oxide phases like cubic MgNiO_2_ and cubic NiAl_2_O_4_, respectively. The Al_2_O_3_-supported Ni catalyst additionally has a crystalline cubic NiO phase. The 5Ni/γAl_2_O_3_ catalyst has all types of reducible NiO species that interact with the supports with different strengths, but the 5Ni/MgO catalyst has a limited amount of reducible NiO species. That means, in comparison to 5Ni/MgO, 5Ni/γAl_2_O_3_ generates a large quantity of catalytic active Ni upon reduction. The smaller particle size is also evident (in the TEM image) over 5Ni/γAl_2_O_3_ than 5Ni/MgO. The IR result and CO_2_-TPD profile of the 5Ni/γAl_2_O_3_ catalyst confirm the inferior interaction of CO_2_ with the catalyst surface than the rest of the catalysts. Both catalysts have cylindrical pore models, but the surface area of 5Ni/γAl_2_O_3_ is also 3.5 times higher than 5Ni/MgO. Altogether, 5Ni/γAl_2_O_3_ has higher contents of reducible NiO species over a larger surface area, and it shows an initial higher H_2_ yield (58%) than the 5Ni/MgO catalyst. However, due to a high coke deposition over 5Ni/γAl_2_O_3_ (weight loss ~76%), the H_2_-yield slows down to 55% (57% CO yield) during 420 min on stream. 5Ni/MgO has various CO_2_-interacting surface species (like carbonate and carboxylate) over the catalyst surface, even under normal environmental conditions. 5Ni/γAl_2_O_3_ has a deficit of such CO_2_-interacting surface species. The CO_2_-TPD profile also showed an enriched basic profile over 5Ni/MgO. The effective interaction of CO_2_ over 5Ni/MgO results in the oxidation of carbon deposits pronounceably. Overall, despite the low content of reducible NiO species and lower surface area, the 5Ni/MgO catalyst has a higher CO_2_ interaction, and it shows a constant ~55 H_2_ yield (58% CO yield) and minimum coke deposition (weight loss 11%) up to 420 min TOS.

Upon the incorporation of MgO in the major alumina supports, mixed-oxide phases (MgAl_2_O_4_ and NiAl_2_O_4_) are organized and individual phases disappear. Mixed-oxide phases are defect-rich and they can induce vacancies and active oxygen formation [37,38] over the catalyst surface. Due to the presence of basic oxides (like MgO), CO_2_ gas interacts more, as well as diss°Ciating over oxide vacancies [39]. Overall, the CO_2_ interaction over the catalyst surface is increased (as verified by the IR and CO_2_-TPD results). The surface area of the 5Ni/20Mg80Al catalyst is improved to 260 m^2^/g (against 196 m^2^/g over 5Ni/γAl_2_O_3_), the pore type of the catalyst changes from cylindrical to plat-like, and mixed-metal-oxide phases (such as cubic NiAl_2_O_4_ and MgAl_2_O_4_) are more organized over the catalyst surface. The charge transition from O^2−^ to Ni^2+^ (°Ctahedral) is also more intensified over the 5Ni/20Mg80Al catalyst than the 5Ni/γAl_2_O_3_ catalyst. The catalytic surface is enriched with CO_2_-interacting surface species and reducible NiAl_2_O_4_ species. Ni derived from NiAl_2_O_4_ is a stabler catalytic active site, and CO_2_-interacting surface species ignites coke deposits effectively. Altogether, the 5Ni/20Mg80Al catalyst shows a constant higher H_2_-yield (61–60%) and CO-yield (70%) and low coke deposit (weight loss ~12%) up to 420 min time on stream. However, it is markable that the CO-yield is pronounceably higher than the H_2_-yield over the 5Ni/20Mg80Al catalyst. It indicates that DRM activity is increased, and a hydrogen-consuming reaction (reverse water gas shift reaction) also prominences over the 5Ni/20Mg80Al catalyst surface. Upon increasing the proportion of MgO (30 wt. %) in the magnesia-alumina supported Ni catalyst (5Ni/30Mg70Al), the reducibility, crystallinity, charge transitions, and thermogravimetry results do not change much. The IR spectra show a relatively intense band for CO_2_-interacting surface species over the 5Ni/30Mg70Al catalyst compared to the 5Ni/20Mg80Al catalyst. That means the 5Ni/30Mg70Al catalyst is more populated with CO_2_-interacting surface species than 5Ni/20Mg80Al. However, the surface area of the catalyst declines by 11%. A relatively lower H_2_ yield (58–57%) up to 420 min time on stream is observed over the 5Ni/30Mg70Al catalyst than the 5Ni/20Mg80Al catalyst. The CO yield (67%) over the 5Ni/30Mg70Al catalyst is again higher than the H_2_-yield. It again indicates that the H_2_-yield of the DRM reaction is affected pronounceably by the RWGS reaction over the 5Ni/30Mg70Al catalyst. Overall, in the mean of the catalytic activity, it is concluded that, among the major alumina (minor magnesia)-supported Ni catalyst, the maximum H_2_-yield is achieved over 5Ni/20Mg80Al. It will be interesting to observe the catalytic activity over major magnesia (minor alumina)-supported Ni catalysts like 5Ni/70Mg30Al and 5Ni/60Mg40Al.

Upon increasing the proportion of magnesia up to 60 wt. % along with alumina as support, the XRD pattern of 5Ni/60Mg40Al is changed from alumina-related phases to magnesia-related phases (like cubic MgO and cubic MgNiO_2_) and surface parameters continue to drop (174.9 m^2^/g surface area, 0.3 cm^3^/g pore volume, and 6.5 nm pore diameter). The reducibility pattern of the catalyst is even now centred around 800 °C, but catalytic active Ni is derived from both the reduction of MgNiO_2_ and “strongly interacted NiO species”. The 5Ni/60Mg40Al catalyst has an intense charge transfer band (from O^2−^ to Ni^2+^). The IR and CO_2_-TPD results confirms the presence of an adequate population of various CO_2_-interacting surface species over the 5Ni/60Mg40Al catalyst. These significant changes in the physio-chemical pattern over the 5Ni/60Mg40Al catalyst result in a prominent rise in catalytic activity. The 5Ni/60Mg40Al catalyst initially shows a 68% H2-yield and 81% CO yield, progressing to a 71% H_2_-yield and 84% CO-yield at 420 min. It is noticeable that the DRM activity progresses with time. It indicates that new active sites may also be generated during the reaction. The activity is enhanced, and the carbon deposit over the catalyst surface is also geared up. The spent-5Ni/60Mg40Al catalyst showed about a 39.09% weight loss. The Raman spectra indicate the presence of higher-intensity defective carbon and graphic carbon bands over the spent 5Ni/60Mg40Al (among spent 5Ni/60Mg40Al, spent 5Ni/70Mg340Al, and spent 5Ni/MgO catalyst systems). That means the deposition of more inert carbon (like defective and graphitic carbon) does not harm the catalytic activity over the 5Ni/60Mg40Al catalyst. It indicates the fine-tuning between the rate of carbon formation (at catalytic active sites) and the rate of carbon diffusion (far from the catalytic active sites) over the 5Ni/60Mg40Al catalyst [40].

In te 5Ni/70Mg30Al catalyst, the surface area of the catalyst drops drastically (surface area 16.8 m^2^/g). The amount of reducible “strongly interacted NiO species” is decreased noticeably. However, the catalyst has the highest population of basic sites and 5Ni/70Mg30Al can interact highest amount of CO_2_-interacting species (confirmed by CO_2_-TPD). The rest of the surface property of Ni/70Mg30Al catalyst is like 5Ni/60Mg40Al catalyst. Altogether, it can be said that the 5Ni/70Mg30Al catalyst has less surface with fewer stable Ni sites than the 5Ni/60Mg40Al catalyst. The 5Ni/70Mg30Al catalyst shows a 64% H_2_ yield and 76% CO yield initially, progressing to a 65% H_2_-yield and 78% CO yield at the end of 420 min. So, in the mean of the catalytic activity, the maximum H_2_-yield, is achieved over the 5Ni/60Mg40Al catalyst.

**Figure 7:**
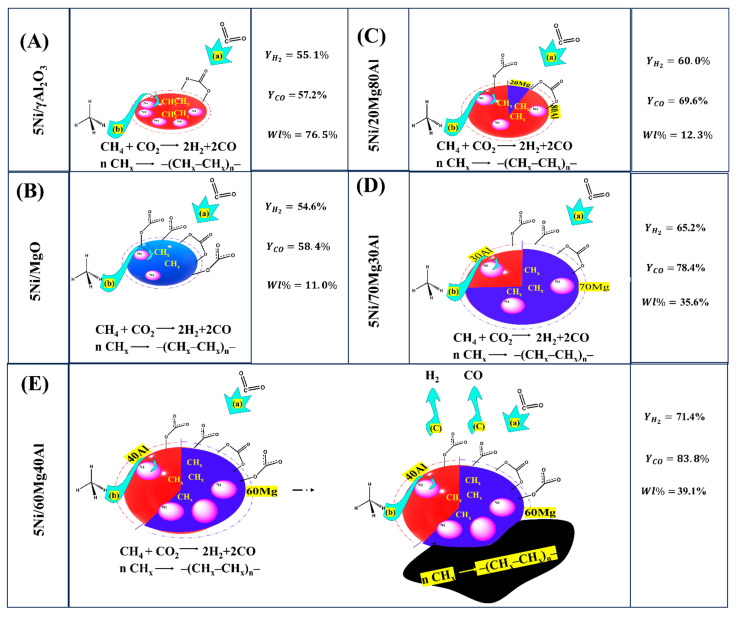
A mechanistic approach over current catalyst systems, (**A**) 5Ni/γAl_2_O_3_, (**B**) 5Ni/MgO, (**C**) 5Ni/20Mg80Al, (**D**) 5Ni/70Mg30Al, and (**E**) 5Ni/60Mg40Al.

Based on the characterization and catalytic activity results, a mechanistic approach can be proposed for the current catalyst system (Figure 7). The reducible species, CO_2_-interacting surface species (or CO_2_ in the gas phase), and timely diffusion of carbon deposits (far from the active sites) are crucial factors in optimizing catalytic activity. Under reductive pretreatment, reducible species generate catalytic active sites Ni, whereas CO_2_ interacts with the surface and forms surface-interacted species (shown by “a” in Figure 7). The active site Ni is responsible for the decomposition of CH_4_ into intermediate CH_x_ (indicated by “b” in Figure 7), whereas CO_2_-interacting species (or CO_2_ in gas phase) oxidize intermediate CH_x_ into H_2_ and CO. The 5Ni/γAl_2_O_3_ surface is enriched with catalytic active sites, which triggers CH_4_ decomposition but a deficit of CO_2_-interacting species allows for CH_x_ polymerization (into coke) more pronounceable than CH_x_ oxidation (into DRM product) (Figure 7A). 5Ni/MgO is populated with CO_2_-interacting species (but fewer Ni sites), which oxidize intermediate CH_x_ promptly. Both catalysts were equally efficient in the mean of H_2_ yield (55%), but 5Ni/MgO had the most minor carbon deposition (Figure 7B). Introducing “alumina along with major MgO in 5Ni/MgO catalyst” or “introducing magnesia with major Al_2_O_3_ in 5Ni/γAl_2_O_3_ catalyst”, both factors (population of reducible species and CO_2_-interacting species) can be balanced. So, the H_2_ yield over the 5Ni/20Mg80Al and 5Ni/70Mg30Al catalysts progressed to 60% and 65%, respectively (Figure 7C,D). The catalytic activity of 5Ni/60Mg40Al needs to be addressed separately. It attains the highest H_2_-yield (71%), as well as the second-highest weight loss (39%) (Figure 7E). The 60:40 magnesia–alumina ratio seems optimum for stabilizing the Ni catalyst toward the DRM reaction, the timely diffusion of coke far from active sites, and exposing catalytic sites for continuous reaction.

## 4. Conclusions

5Ni/γAl_2_O_3_ has an enriched reducibility, whereas the 5Ni/MgO catalyst has a rich basic profile for CO_2_ interaction. At the end of 420 min time on stream, the earlier one shows a ~55% H_2_ yield with a huge coke deposit (weight loss of ~76%), but the latter has the same activity with a minimum coke deposit (weight loss of 11%). Upon 20 wt. % incorporation of the basic MgO into the acidic Al_2_O_3_ support, the expanded surface of the 5Ni/20Mg80Al catalyst is populated with stable Ni sites (derived from NiAl_2_O_4_) and a variety of CO_2_-interacting surface species. The catalytic activity over 5Ni/20Mg80Al progresses to a ~61% H_2_ yield with a relatively good coke resistance (weight loss ~12%) up to 420 min time on stream. Further incorporation of the basic magnesia into alumina, as for the 5Ni/30Mg70Al catalyst case, shows no beneficial effect on DRM. On the other hand, the catalytic activity is significantly enhanced when the support is made largely by magnesia (upon incorporating a large amount of magnesia with alumina) like 5Ni/60Mg40Al. The 5Ni/60Mg40Al catalyst has magnesia and mixed-magnesia-related phases (MgO, MgNiO_2_) and the catalytic active sites over the catalyst are derived by reduction of the MgNiO_2_ phase and “strongly interacted NiO species”. The 5Ni/60Mg40Al showed the highest hydrogen yield (~71%) at the end of 420 min, although it possesses this even against a high carbon deposit (weight loss 39%). Upon increasing the proportion of Mg in support further (5Ni/70Mg30Al), the surface area drops suddenly, and relatively fewer stable Ni sites are nurtured, which results in more inferior activity over 5Ni/70Mg30Al than 5Ni/60Mg40Al catalyst. There are three clear outcomes from the current research.

(1) Ni dispersed over magnesia-alumina with weight ratios of 7/3 and 3/7 has a strong suppression of carbon deposit.
(2) Ni dispersed over magnesia-alumina with weight ratios of 2/8 and 7/3 have adequate amounts of reducible species and CO_2_-interactive species, which confirms high activity (>60% H_2_-yield)
(3) Ni dispersed over magnesia-alumina with a weight ratio of 6/4 has a better coke diffusion mechanism, where active sites remain exposed and convey a > 70 H_2_-yield.

## Figures and Tables

**Table 1:** Surface area, pore volume, and pore diameter of 5Ni/MgO, 5Ni/γAl_2_O_3_, and 5Ni/xMg(100 − x)Al (x = 20, 30, 60, and 70 wt. %) catalyst.

Catalyst Sample	Surface Area (m^2^/g)	Pore Volume (cm^3^/g)	Diameter (nm)
5Ni/MgO	55.2	0.5	35.5
5Ni/70Mg30Al	16.8	0.1	16.0
5Ni/60Mg40Al	174.9	0.3	6.5
5Ni/γAl_2_O_3_	195.5	0.6	9.7
5Ni/20Mg80Al	260.2	0.5	7.1
5Ni/30Mg70Al	230.1	0.5	7.0

## Data Availability

Data is contained within the article.

## Supplementary Materials

The following supporting information can be downloaded at: https://www.mdpi.com/article/10.3390/nano13232984/s1, Figure S1. Pore size distribution plot of (a) 5Ni/MgO; (b,c) 5Ni/xMg(100 − x)Al (x = 70, 60); (d) 5Ni/γAl_2_O_3_; (e,f) 5Ni/xMg(100 − x)Al (x = 20, 30).
