# Supplementary material for: Alumina-Magnesia-Supported Ni for Hydrogen Production via the Dry Reforming of Methane: A Cost-Effective Catalyst System
Source: Nanomaterials (Basel). 2023 Nov 21;13(23):2984. doi: 10.3390/nano13232984 (PMC10708042; doi:10.3390/nano13232984)
Supplement: Supplementary file 1 [file nanomaterials-13-02984-s001.zip › nanomaterials-2718915-supplementary.pdf]

Supplementary Materials:

Alumina-Magnesia-Supported Ni for Hydrogen Production via the Dry Reforming of Methane: A Cost-Effective Catalyst System

Abdulaziz A. M. Abahussain [1], Ahmed S. Al-Fatesh [1,*], Naitik Patel [2], Salwa B. Alreshaidan [3], Nouf A. Bamatraf [3], Ahmed A. Ibrahim [1], Ahmed Y. Elnour [1], Jehad K. Abu-Dahrieh [4,*], Ahmed E. Abasaeed [1], Anis H. Fakeeha [1] and Rawesh Kumar [2]

1 Chemical Engineering Department, College of Engineering, King Saud University, P.O. Box 800, Riyadh 11421, Saudi Arabia; a.abahussain@ksu.edu.sa (A.A.M.A.); aidid@ksu.edu.sa (A.A.I.); aelnour@ksu.edu.sa (A.Y.E.); abasaeed@ksu.edu.sa (A.E.A.); anishf@ksu.edu.sa (A.H.F.)

2 Department of Chemistry, Indus University, Ahmedabad 382115, Gujarat, India; naitikvagdoda@gmail.com (N.P.); kr.rawesh@gmail.com (R.K.)

3 Department of Chemistry, Faculty of Science, King Saud University, P.O. Box 800, Riyadh 11451, Saudi Arabia; chem241@ksu.edu.sa (S.B.A.); 442204210@student.ksu.edu.sa (N.A.B.)

4 School of Chemistry and Chemical Engineering, Queen's University Belfast, Belfast BT9 5AG, Northern Ireland, UK

* Correspondence: aalfatesh@ksu.edu.sa (A.S.A.-F.); j.abudahrieh@qub.ac.uk (J.K.A.-D.)

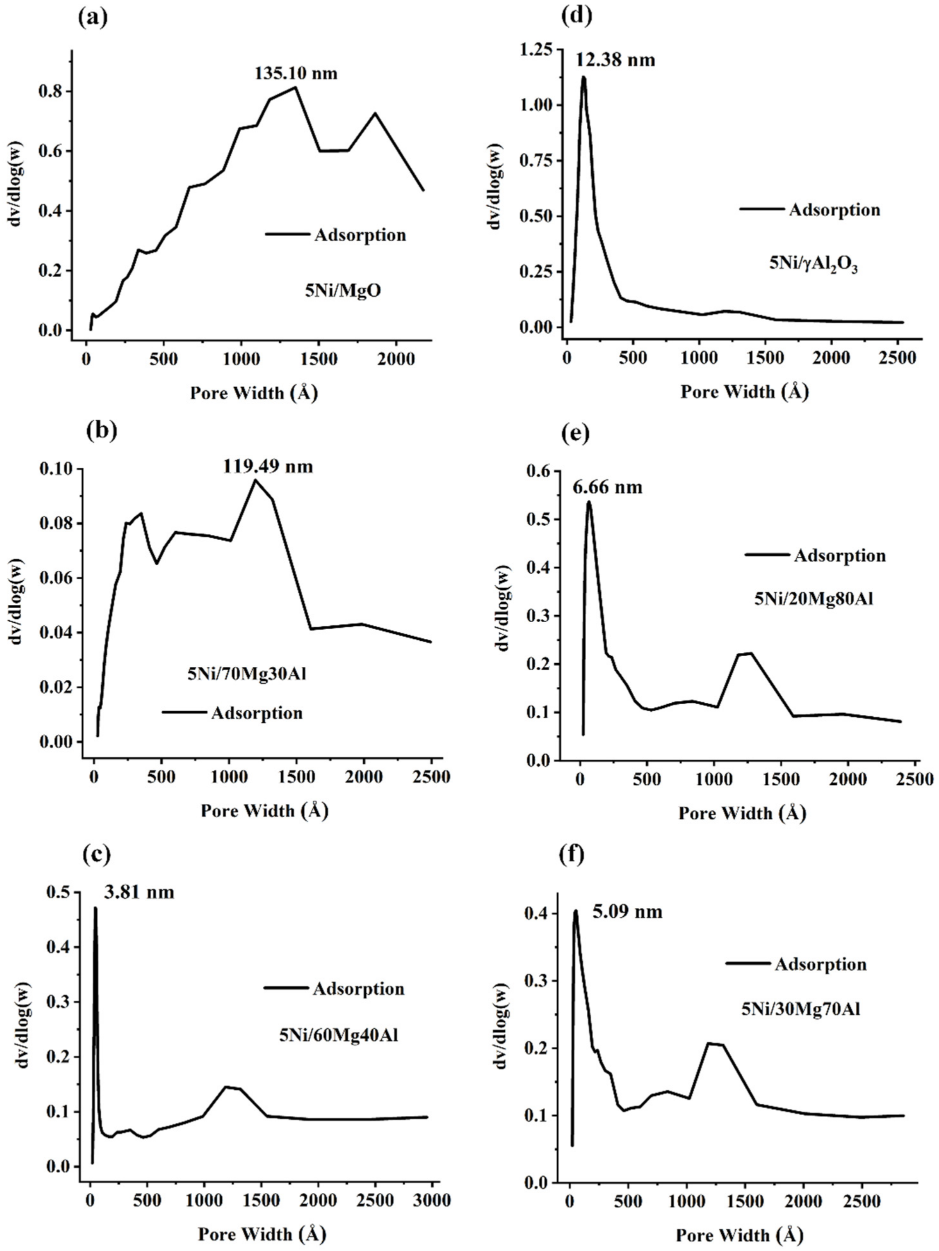

Figure S1. Pore size distribution plot of (a) 5Ni/MgO; (b–c) 5Ni/xMg(100-x)Al (x = 70,60); (d) 5Ni/γAl_2O_3; (e–f) 5Ni/xMg(100−x)Al (x = 20,30).
